# Predicting Failure of Additively Manufactured Specimens with Holes

**DOI:** 10.3390/ma16062293

**Published:** 2023-03-13

**Authors:** Gina Eileen Chiara Schmeier, Clara Tröger, Young W. Kwon, Delf Sachau

**Affiliations:** 1 Department of Mechanical & Aerospace Engineering, Naval Postgraduate School, Monterey, CA 93943, USA; 2 Fakultät Maschinenbau, Helmut-Schmidt-Universität/Universität der Bundeswehr, 22043 Hamburg, Germany

**Keywords:** additive manufacturing, perforated specimen, failure load, combined loading, failure criterion

## Abstract

Experimental and computational studies were conducted to predict failure loads of specimens containing different-sized holes made using the additive manufacturing (AM) technique. Two different types of test specimens were prepared. Flat specimens, manufactured from polylactic acid (PLA), were subjected to uniaxial loading. Tubular specimens, made of polycarbonate (PC), were subjected to combined loading that was applied using uniaxial testing equipment. Test specimens were uniquely designed and printed to apply the combined bending and torsional loads to tubular specimens. A newly developed failure theory was applied to predict the loads that would result in the fracture of these test specimens. This theory is composed of two conditions related to stress and the stress gradient to be simultaneously satisfied to predict failure. The failure loads predicted using the new failure criteria were compared closely with the experimental data for all test specimens. In addition, a semi-empirical equation was developed to predict the critical failure surface energy for different printing angles. The critical failure surface energy is a material property and is used for the stress gradient condition. Using the semi-empirically determined values for the failure criterion provided close agreement with experimental results.

## 1. Introduction

Additively manufactured, also known as 3D printed, parts are being used more frequently in a diverse set of applications. When the user-friendly technology of 3D printers is combined with polymer substrates, it became possible for an even greater body of researchers to produce parts for a wider variety of applications. As a result of this greater usage, understanding the behavior of parts produced in this way has become increasingly important. Compared to significantly heavier materials, polymer composites have a high stiffness and strength relative to their weight. This is a great advantage, especially for endeavors that depend on high-strength, low-weight materials. In the aerospace industry, for example, weight-saving components are increasingly being implemented using 3D printed polymers. Airbus relies on AM to reduce the weight of parts contained in the Racer high-speed helicopter [1].

Because the cost of 3D printers has dropped significantly in recent years, the technology has also become increasingly well known among private users who are inexpensively producing smaller models. A large variety of printable materials has also become accessible. Most printing is conducted using polymers such as polylactic acid (PLA), polycarbonate (PC), and acrylonitrile butadiene styrene (ABS), but metals that melt at low temperatures can also be used. Metals with a higher melting point and composites are also used as substrate materials, although they are currently only available at a very high price [2].

With the expansion of applications using AM, the question arises as to whether they are strong and durable enough to be used as load-carrying members. As a result, it is becoming increasingly important to understand the failure of these structures under various loading conditions. To address this question, research has been conducted to understand the strength and stiffness of 3D printed parts [3,4,5,6,7,8,9,10,11,12]. Those studies investigated the effect of various printing parameters on the strength and stiffness of 3D printed parts. Because most practical parts include geometric features that result in stress concentrations, this research focused on investigating the change in failure loading on samples containing a circular hole.

Having the ability to predict failure loads of such parts is necessary to better design 3D printed parts. Having a reliable theory that allows the estimation of failure before it occurs is a necessity. Many different failure criteria have been proposed, but they vary depending on whether the material of interest is isotropic or anisotropic, brittle, or ductile. Furthermore, different failure criteria have also been used depending on the shape of a defect such as notches in a part.

For specimens containing geometric features that induce a local stress concentration like a hole, a variety of failure criteria have been proposed [13,14,15,16,17]. Those failure criteria use a concept of characteristic critical distance. In other words, failures are predicted on the basis of stresses at critical distances or the average stresses up to critical distances. Different failure criteria have proposed different ways to determine the critical distances. More recently, a new unified failure criterion was developed [18,19,20]. The previous study demonstrated that the new failure criterion applies to brittle materials regardless of whether they contain a defect or not.

Even though there have been many studies on 3D printed parts, there has been no analytical attempt to predict the failure of 3D printed parts with notches, to the best of the authors’ knowledge. Thus, the objective of this study was to experimentally measure failure loads of various 3D printed flat and tubular specimens containing different sizes of circular holes and to predict the failure loads using the new failure criterion. The flat specimens were made of PLA and subjected to uniaxial tensile loading, while tubular specimens were made of PC and subjected to combined loading. Because PLA specimens were stronger and stiffer along the printing direction, i.e., raster angle, they behaved as a fibrous composite [9,11]. As the raster angle varied, the PLA specimens behaved as laminated fibrous composites. One difference is that PLA specimens are made of one material while fibrous composites are made of fiber and matrix materials. This study evaluated how well the new failure criterion could predict failure loads for different kinds of 3D printed specimens subjected to uniaxial and combined loading, respectively.

To apply combined loading using a uniaxial testing machine, a new design of testing setup and test specimens was developed for this study, which took advantage of the flexibility of 3D printing. Combined loading can be applied by testing equipment that has a special testing machine to apply tensile and torsional loads simultaneously. To use a uniaxial testing machine for multiaxial loading, additional test setups were introduced. Those setups typically apply biaxial loading [21]. In this study, both bending and torsional loads were applied together as a combined load using uniaxial testing equipment.

The next section describes the preparation of 3D printed specimens and the design of combined loaded test specimens. Subsequent sections describe the material testing and an investigation of the failure criteria [18,19,20], followed by computational modeling results. Lastly, predictions of failure are made using the new failure criterion.

## 2. Preparation of 3D Printed Specimens

**Figure 1:**
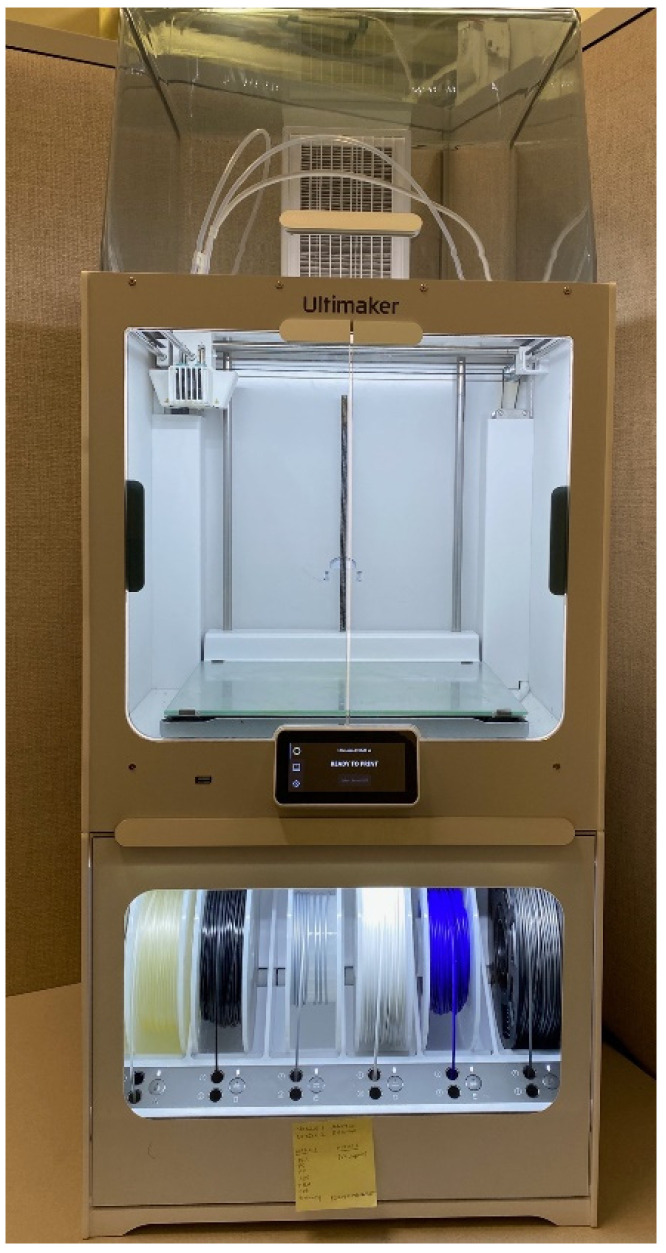
Image of Ultimaker© S5 printer.

All the specimens were modeled using the program called Solidworks and converted into STL files using the default setting in Solidworks. Then, the specimens were fabricated and prepared using the fused filament fabrication (FFF) printing technique with different polymer filaments. PLA was selected for the flat, rectangular, test specimens because its properties can be changed by altering printing parameters, such as direction. An Ultimaker© S5 printer (Utrecht, Netherlands), which is shown in Figure 1, was used to print all of the specimens tested under this effort. The printer has a build dimension of 330 × 240 × 300 mm, and it has a range of resolution of 0.25 mm to 0.8 mm. Its nozzle temperature is between 180 °C and 280 °C. The nozzle can be heated up in less than 2 min.

**Figure 2:**
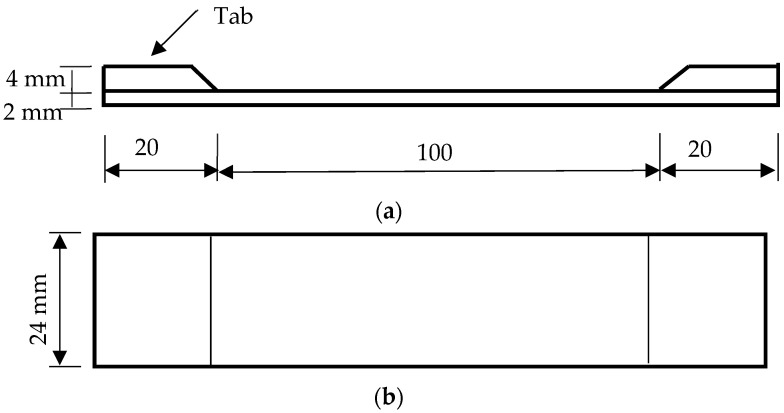
Rectangularly shaped test specimens: (**a**) side view; (**b**) top view.

**Figure 3:**
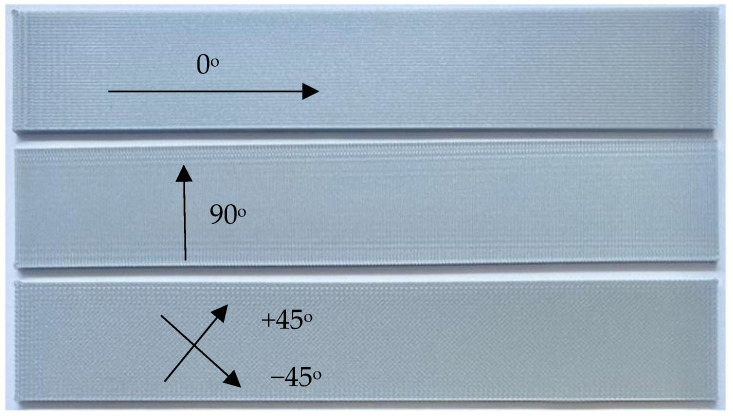
Test specimen different printing orientations.

All the filaments used in this study were commercially available, and their material specifications are provided on their respective websites [22]. In this study, the PLA specimens were designed to produce orthotropic specimens with properties along the printing direction being measurably different from those measured in its orthogonal direction. To achieve that, the print settings shown in Table 1 were used to print all PLA samples. Every specimen was printed with the setting of 100% infill with a rectangular geometry measuring 140 mm long by 24 mm wide by 2 mm thick in the test section. As the specimens were held by the grips, the gauge length was set to be 100 mm as sketched in Figure 2. The width of specimens was chosen to fit the grip width of the test equipment, and the printing direction was varied from one sample to the next. Filament printing orientations were 0°, 90°, or +q/−q, where q was chosen to be 30°, 45°, or 60°. Figure 3 shows the different print directions measured relative to the direction of loading, with 0° corresponding to the axis along which the load was applied.

**Figure 4:**
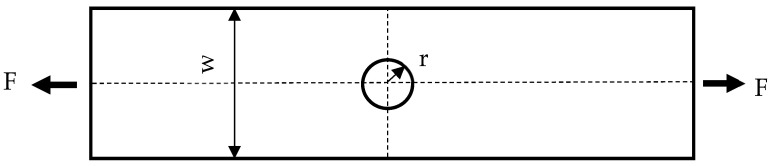
Center hole test specimen with 2 mm thickness (W = 24 mm, r = 1.5, 3, or 4.5 mm).

Two types of PLA specimens were printed in this manner. One type of specimen was printed with a center hole while the other was printed without a hole. The latter specimens were used to determine the material properties of PLA specimens, while the former ones were used to measure how the failure load was influenced by such a geometric feature. Three to five specimens were printed and tested for every rectangular specimen to check the repeatability of test data. Because the test data were quite consistent, additional specimens were not printed and tested. The rectangular specimens without holes were printed with tabs for the grip sections of the specimen as one single piece as sketched in Figure 2. This was to ensure that failure occurred at the specimen midsection rather than at the grip sites. To simplify printing the samples, the tabs were printed on only one side of the specimen rather than both sides as that would have required the use of printing support material. Upon testing, this method was shown to not affect the overall test results. The specimens printed with center holes did not require tabs because a failure occurred at the minimum cross-section in the hole. The hole size was 3 mm, 6 mm, or 9 mm in diameter as shown in Figure 4. Three to five specimens of each type were printed to ensure enough duplicates for testing.

**Figure 5:**
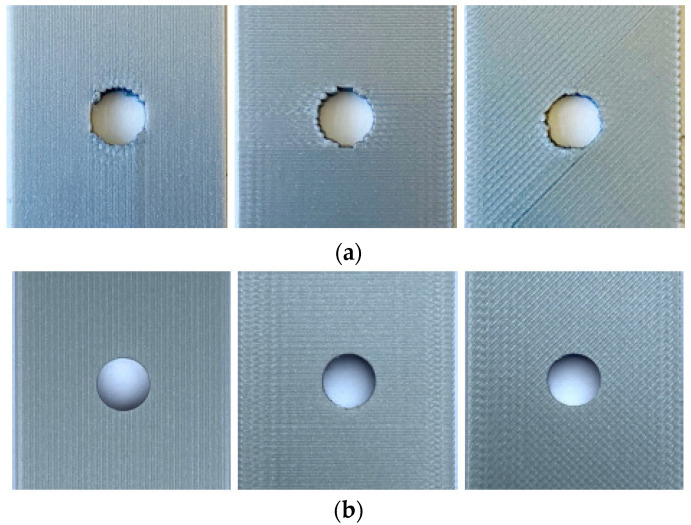
The 6 mm hole diameter samples: (**a**) PH6.0 (**left**), PH6.90 (**middle**), and PH6.45 (**right**); (**b**) DH6.0 (**left**), DH6.90 (**middle**), and DH6.45 (**right**).

Two different techniques were used to generate the center hole. In one set of samples, it was printed in, while, in the other, the hole was drilled out. The former specimens were denoted by PHx.y (printed hole) while the latter specimens were called DHx.y (drilled hole), where x indicates the hole diameter, and y indicates the printing angle +q°/−q°. Figure 5 shows that there is a difference in quality along the edge of the hole based on the chosen manufacturing technique. Figure 5a shows the printed holes in the specimens using different print angles, while Figure 5b shows the drilled holes with the same orientations. Because the printed hole did not have a smooth circular edge, some portions of the edges were filed down to make them smooth. The drilling produced much smoother edges of the holes as compared in Figure 5. The specimens with drilled holes were modeled for predicting their failure stresses because smoother hole edges are much easier to model than rough edges.

**Figure 6:**
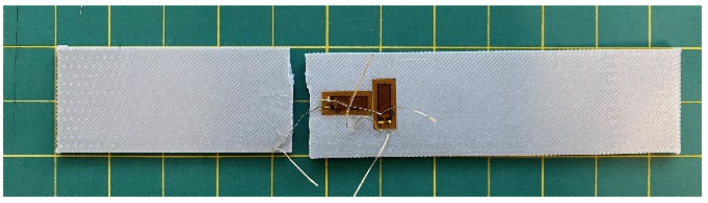
Post-test photograph of a specimen without a circular hole.

All the tests were undertaken using an Instron^© 2023^ uniaxial test machine set at a crosshead speed of 2 mm/min. The first set of tests was performed on the rectangular specimens without a hole. Later, the samples with 0°, 90°, and ±45° raster angles were tested to determine their strength and stiffness. Some of those samples had strain gauges in the longitudinal and transverse directions to measure Poisson’s ratio. Figure 6 is a post-test photograph of an instrumented test specimen. The tabs are not visible, however, because they are on the back side of the specimen.

Once the stiffness and strength of the PLA specimens were obtained for the specimens without holes, the specimens with holes were tested until fracture using the same testing condition and equipment. This showed a reduction in failure loads resulting from the holes, which is further discussed later.

## 3. Combined Loading Specimen Design

A new design was developed for conducting combined loading of test specimens using a standard uniaxial testing machine. The new designs were printed using AM because it affords researchers greater design flexibility than that offered using traditional machining methods.

**Figure 7:**
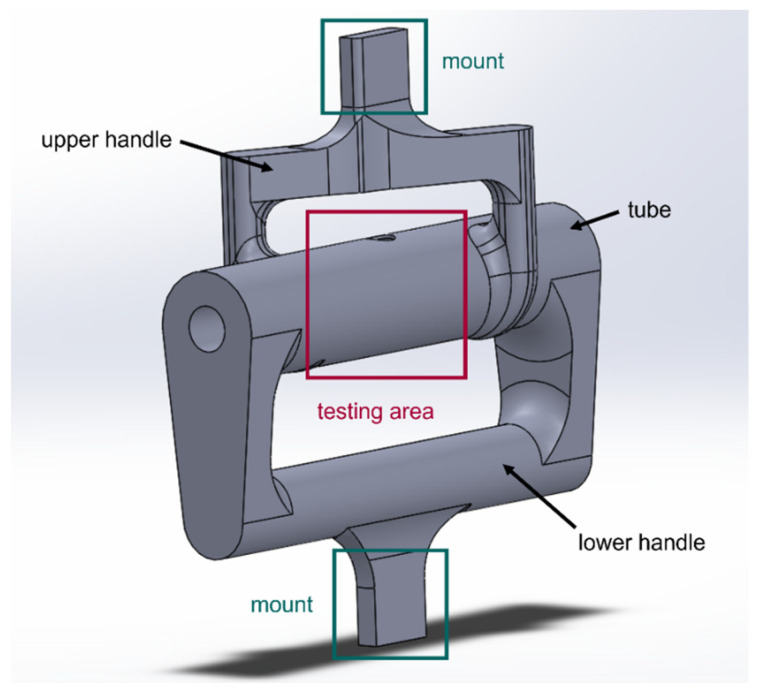
One-piece combined loading tubular specimen design.

Several different combinations of combined loading are possible. They might consist, for example, of biaxial loads, or a combination of tension and torsion among numerous other possibilities. In this study, the new test specimen was designed to simultaneously apply bending and torsion. The initial design of the combined loading specimen was a single-piece design as shown in Figure 7. To decrease printing times, parts of the design were hollow. The design consists of three portions: upper and lower handles, and a tubular test section. The lower handle is aligned with the testing section in the same plane while the upper handle is out of the plane of the test section. As the mount sections of both upper and lower handles are pulled apart in the uniaxial test machine, the test section, which may or may not have a hole, experiences both bending and torsion. If the upper handle is not out of the plane and aligned with the test section like the lower handle, the test section would be subjected to a bending load like the four-point bending test. The out-of-plane offset of the upper handle results in torsional loading.

**Figure 8:**
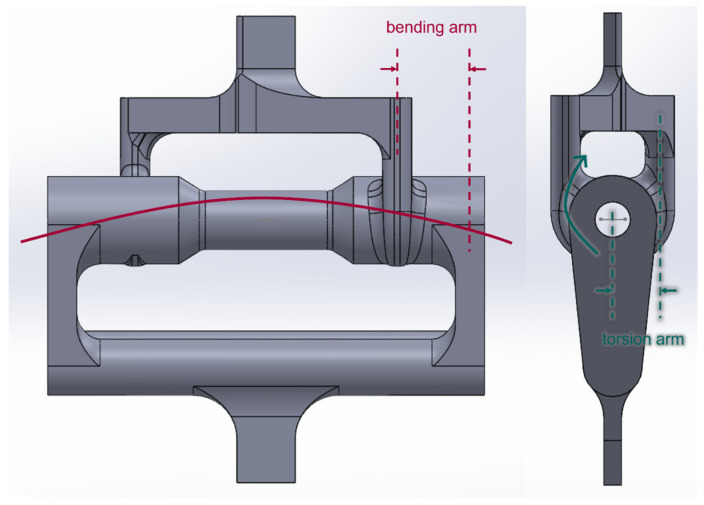
Initial combined bending and torsion design.

To increase the torsion experienced by the sample, the offset distance of the upper handle was increased while the bending load was controlled by altering the distance between the upper and lower handles as seen in Figure 8. As the test specimen was subjected to axial loading *F*, the simplified theory was used to estimate resultant stresses. A numerical analysis using finite element analysis (FEA) was also conducted to determine the stresses experienced by the specimen. The bending stress at the outer surface of the test section is computed using Equation (1).

(1) $$\sigma_{b}=\frac{Mr_{o}}{I_{b}},$$

where the bending moment *M* is calculated by

(2) $$M=\frac{FL_{b}}{2},$$

where $L_{b}$ is the bending arm shown in Figure 8, and $I_{b}$ is the second moment of inertia of the test cross-section, calculated as

(3) $$I_{b}=\frac{\pi }{4}\left(r_{o}^{4}-r_{i}^{4}\right),$$

where $r_{o}$ and $r_{i}$ are the outer and inner radii of the test section.

The shear stress resulting from the torsional loading is computed as

(4) $$\tau_{t}=\frac{Tr_{o}}{J}.$$

The polar moment of inertia *J* is twice the value of the second moment of inertia $I_{b}$. The torsional moment *T* is calculated from

(5) $$T=\frac{FL_{t}}{2},$$

where $L_{t}$ is the torsional arm as sketched in Figure 7. Lastly, the maximum normal stress of the combined stress state is expressed as

(6) $$\sigma_{\max}=\frac{\sigma_{b}}{2}+\sqrt{\frac{\sigma_{b}^{2}}{4}+\tau_{t}^{2}}.$$

In a later section, these simplified analytical solutions are compared to the FEA results.

In the initial design, both the upper and the lower handles, as well as the test specimen, were printed as a single piece using a PC material. When tested, failure occurred at either the upper or the lower handle. Although FEA indicated that failure should occur at the test section because the handles were so much thicker and, hence, believed to be much stronger, this was not the case. The reason for such an unexpected failure was believed to be residual stresses in handle sections. In one instance of a one-piece specimen, a crack occurred in the lower handle during the 3D printing process because of residual thermal stress. In addition, printing the single-piece design presented other difficulties, e.g., taking longer and requiring more material.

**Figure 9:**
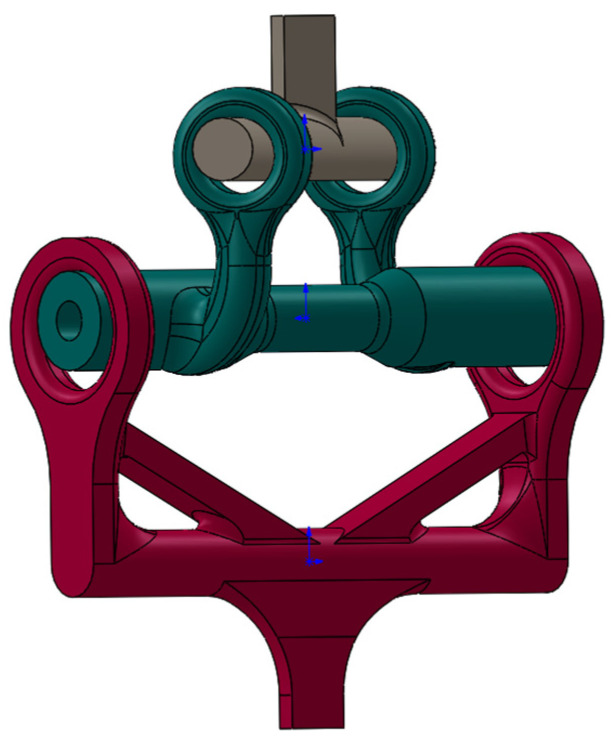
Three-part design: upper handle, test tube, and lower handle.

To overcome the difficulties, the initial design was modified. The lower and upper handles, as well as the test specimens, were printed individually. The three parts of the modified design are shown in Figure 9. The lower and upper handles were printed out of an aluminum alloy to be deliberately stronger than the test section. Once both handles were printed, they could be used repeatedly unless major changes are required in the specimen design. The new design eliminated potential failure in the upper and lower handles guaranteeing failure at the test section of interest. Printing out test specimens in this manner also saved appreciable time and material.

**Figure 10:**
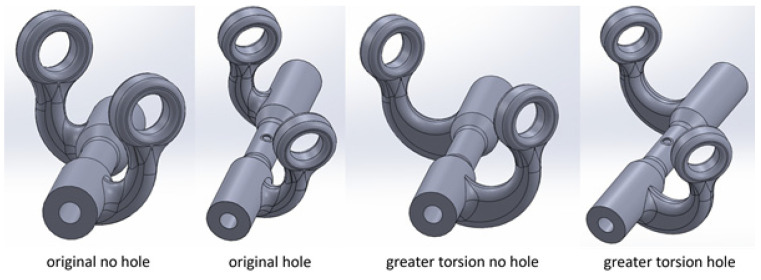
Different test specimens.

The test specimens for combined loading were printed using two different 3D printers. The Ultimaker© S5 was again used to print a black PC at 100% infill. These settings are listed below in Table 2. This setting was determined on the basis of the recommended temperature of the PC material by the manufacturer. The other printer was a Fortus© 450mc manufactured by Stratasys using a white PC. This printer is industrial AM equipment and can have a layer height from 0.127 mm to 0.330 mm with a part accuracy of ±0.0015 mm/mm. The default setting was used for the Fortus© 450mc printer. Using each printer, four different types of test specimens were fabricated as shown in Figure 10. Two of the test specimens had a longer torsional arm than the others. Of the two specimens with otherwise identical torsional arms, one had a 6 mm diameter hole and the other had no hole at all. The difference between the two 3D printers was the wall thickness of the test section. The test specimens made from Ultimaker© were 0.8 mm thick, while the test section manufactured using the Fortus© printer was 1.0 mm thick.

## 4. Testing and Results

**Figure 11:**
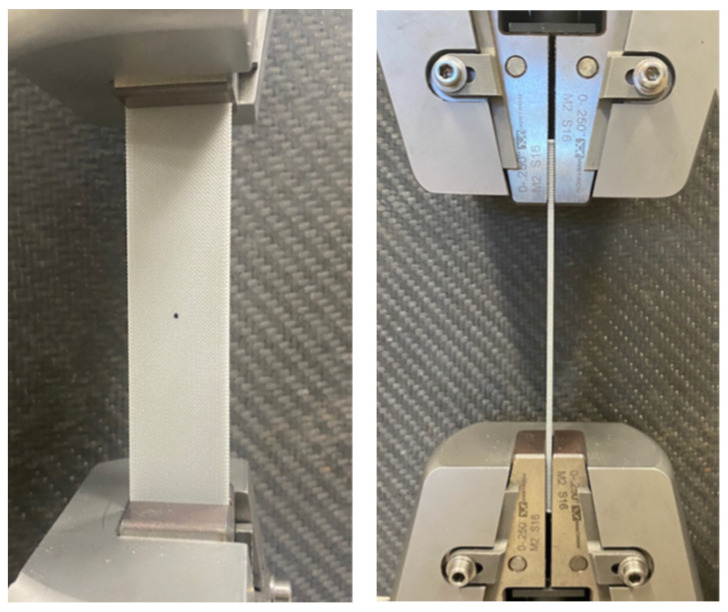
A rectangular test specimen with a hole held by the testing equipment.

**Figure 12:**
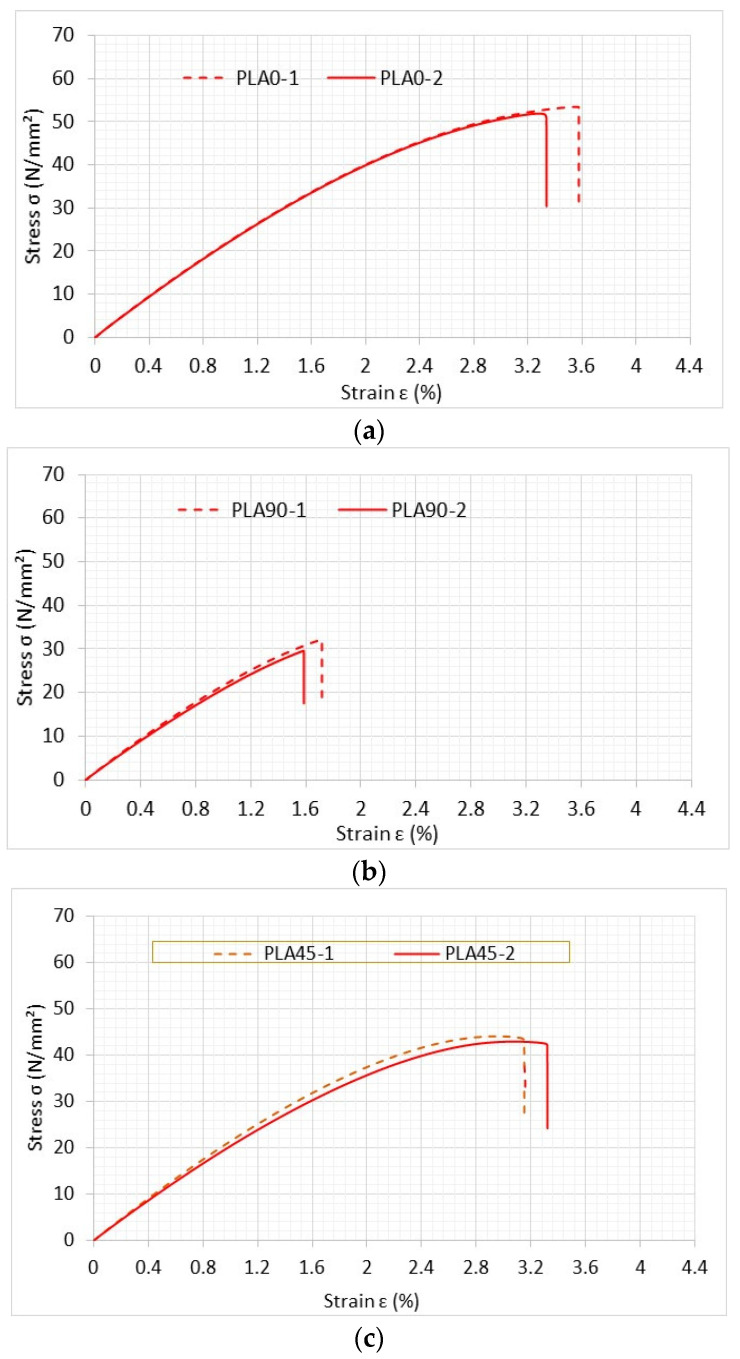
Stress–strain curves of PLA specimens with different printing angles: (**a**) 0°; (**b**) 90°; (**c**) ±45°.

All the tests were conducted using Instron following ASTM D638 as close as possible. Figure 11 shows a rectangular specimen with a hole installed at the grip of the testing equipment. Tensile tests of PLA specimens were first conducted on the specimens without any holes in the central section. The stress–strain curves of three test samples with different print angles are shown in Figure 12. The strength and stiffness of these specimens were different, depending on the printing angle. When the printing angle aligned with the loading direction, as it did in the case of the 0° specimen, the samples were the strongest and stiffest of any printing direction. This was a good indication that PLA specimens were orthotropic and possessed properties that have a strong correlation with the printing angle [9,11]. The longitudinal and transverse elastic moduli and tensile strength were obtained from the 0° and 90° specimens. The ±45° specimen was used to determine the shear modulus as described below.

The stress–strain constitutive matrix for an orthotropic material can be expressed as

(7) $$\left\{\begin{array}{c} \varepsilon_{1}\\ \varepsilon_{2}\\ \gamma_{12} \end{array}\right\}=\left[\begin{array}{ccc} S_{11} & S_{12} & 0\\ S_{12} & S_{22} & 0\\ 0 & 0 & S_{33} \end{array}\right]\left\{\begin{array}{c} \sigma_{1}\\ \sigma_{2}\\ \tau_{12} \end{array}\right\},$$

where ε and γ are normal and shear strains, while σ and τ are the normal and shear stresses. The matrix coefficients $S_{ij}$ are determined using the following expressions:

(8) $$S_{11}=\frac{1}{E_{1}},S_{12}=-\frac{\nu_{12}}{E_{1}}=-\frac{\nu_{21}}{E_{2}},S_{22}=\frac{1}{E_{2}},S_{66}=\frac{1}{G_{12}},$$

where $E_{1}$ and $E_{2}$ were determined from the slope generated from test results of 0° and 90° specimens, respectively. Because the stress–strain curves have nonlinear sections, the initial part of the curves was used to determine the elastic moduli. Poisson’s ratio $\nu_{ij}$ was also determined from the strain-gauge readings. Those material properties were also used later for computer modeling.

To determine the shear modulus $G_{12}$, a stress–strain transformation was used. When the printing angle is oriented at an angle q, the transformed equation becomes

(9) $$\left\{\begin{array}{c} {\bar{\varepsilon }}_{1}\\ {\bar{\varepsilon }}_{2}\\ {\bar{\gamma }}_{12} \end{array}\right\}=\left[\begin{array}{ccc} {\bar{S}}_{11} & {\bar{S}}_{12} & {\bar{S}}_{13}\\ {\bar{S}}_{12} & {\bar{S}}_{22} & {\bar{S}}_{23}\\ {\bar{S}}_{13} & {\bar{S}}_{23} & {\bar{S}}_{33} \end{array}\right]\left\{\begin{array}{c} {\bar{\sigma }}_{1}\\ {\bar{\sigma }}_{2}\\ {\bar{\tau }}_{12} \end{array}\right\},$$

in which the superimposed bar denotes the values in the new coordinate system oriented with an angle q with respect to the original coordinate system. The first component in Equation (9) is expressed as

(10) $${\bar{S}}_{11}=S_{11}\cos^{4}\theta +\left(2S_{12}+S_{33}\right)\sin^{2}\theta \cos^{2}\theta +S_{22}\sin^{4}\theta .$$

When q is ±45°, Equation (10) is simplified to

(11) $$4{\bar{S}}_{11}=S_{11}+\left(2S_{12}+S_{33}\right)+S_{22},$$

where ${\bar{S}}_{11}$ is obtained from the slope of the stress–strain curve of the ±45° specimen, and $S_{11}$, $S_{22}$, and $S_{12}$ are obtained from 0° and 90° specimens. Thus, $S_{33}$ is determined using Equation (11), and the shear modulus $G_{12}$ is computed using Equation (8). All the material properties were measured and computed from PLA test specimens without holes. These are listed in Table 3 below.

**Figure 13:**
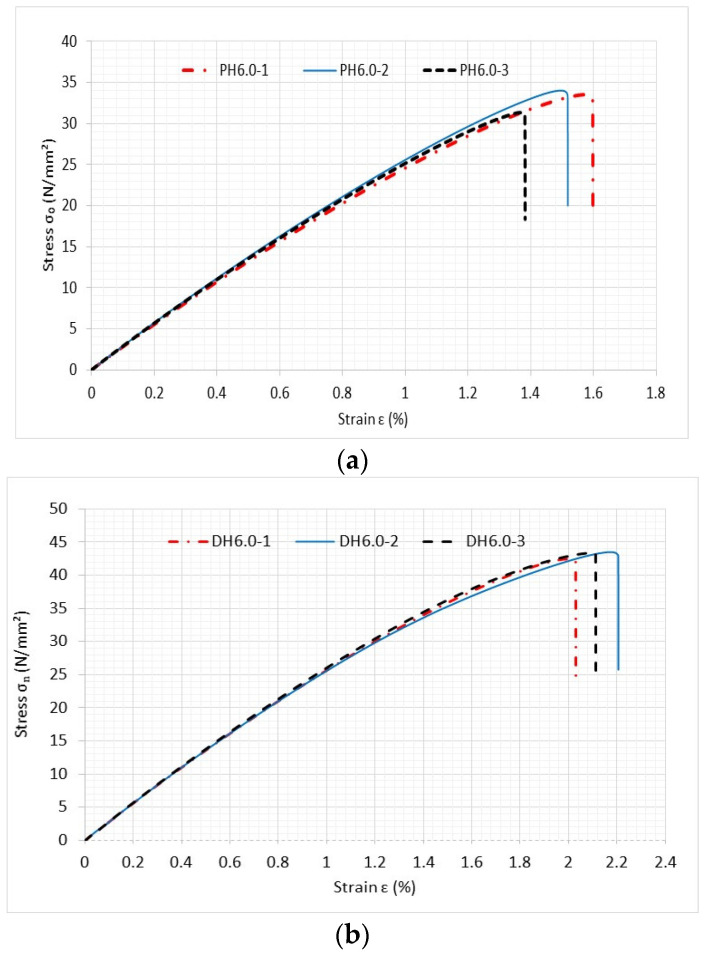
Comparison of stress–strain curves of specimens with (**a**) printed hole and (**b**) drilled hole with 6 mm diameter and 0° print angle.

The next set of tests was conducted using perforated PLA specimens to compare printed holes to drilled ones. Figure 13 compares the stress–strain curves of both specimens with printed and drilled holes, both of which measured 6 mm in diameter. The printing angle was 0° for the specimens shown in Figure 13.

The specimens with printed holes consistently exhibited lower failure stresses than those with drilled holes. Similar comparisons were also made to other specimens with different sizes of holes and printing angles. The specimens with drilled holes consistently exhibited greater strength than those with the printed ones. The main cause of this behavior was due to the smoother edges that the drilled holes had versus the jagged holes that occurred when they were printed as previously shown in Figure 5. Roughness at the edges of the printed holes resulted in a reduction in failure strength. It is important to point out that all the comparisons with analytical predictions were made using the drilled hole test specimens so that any hole roughness could be safely neglected in the FEA modeling.

**Figure 14:**
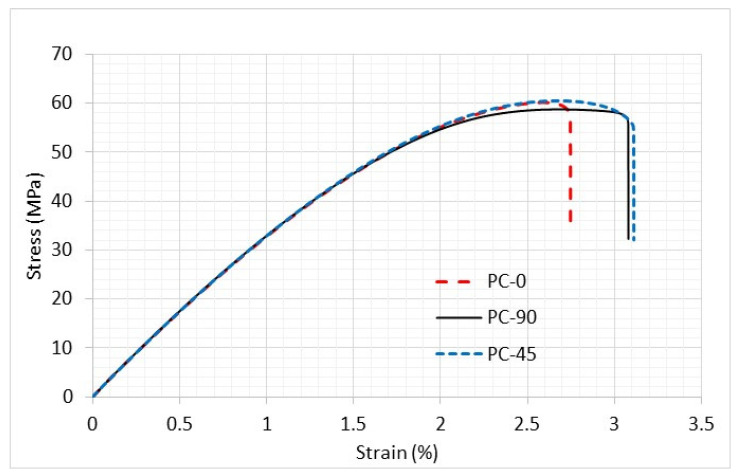
Stress–strain curve of PC specimens without a hole and different printing angles.

PC specimens were tested in identical geometries using tabs just like the previous PLA samples. Initial testing was conducted on PC samples without holes to determine their strength and stiffness. The same three printing angles, 0°, 90°, and ±45°, were used and are plotted below in Figure 14.

The stress–strain curves of these PC specimens were, however, almost identical regardless of which printing angle was used. This suggests that PC specimens should be considered as an isotropic material rather than an orthotropic one. The elastic modulus was 3.3 GPa, and a Poisson’s ratio of 0.375 was obtained from the use of two perpendicular strain gauges.

## 5. Failure Criteria

The recently proposed unified failure criterion consists of two parts, both of which must be satisfied for failure to occur [18,19,20]. The failure criterion can be applied to specimens regardless of whether they have a defect, e.g., a crack or a hole. The first part is expressed as

(12) $$\left|\sigma_{eff}\right|\geq \left|\sigma_{fail}\right|,$$

where $\sigma_{eff}$ is the effective stress, and $\sigma_{fail}$ is the failure strength of the material. For brittle materials, the maximum or minimum normal stress is used as the effective stress depending on tension or compression, and the failure strength is obtained from a uniaxial tensile or compressive test.

The second part is based on the stress gradient. The criterion is expressed as

(13) $$\sigma_{eff}\geq {\left(\left|\frac{d\sigma_{eff}}{ds}\right|E\kappa_{fail}\right)}^{\frac{1}{3}},$$

in which $\left|\frac{d\sigma_{eff}}{ds}\right|$ is the stress gradient and *s* is along the path of the failure. *E* is the elastic modulus, and $\kappa_{fail}$ is another failure value that has the same units as the critical energy release rate.

To predict failure, both Equations (12) and (13) must be satisfied. Usually, one of the two dominates depending on the geometry. The two failure values, $\sigma_{eff}$ and $\kappa_{fail}$, are needed to predict failure. Henceforth, the former is called failure strength while the latter is referred to as critical failure surface energy. To determine the failure strength and critical failure surface energy, unnotched and notched specimens were tested, and the respective values were measured. Then, those failure values were used for any specimen, regardless of sizes, shapes, or locations of notches, if it was the same material.

All the specimens with a circular hole in this study demonstrated that Equation (13) was the more limiting condition of the two. In other words, the effective stress at the initiation location was greater from Equation (13) than that from Equation (12). Therefore, the critical surface failure energy was used to predict failure stresses at notches. Because both PLA and PC are somewhat brittle, the maximum normal stress, or the largest principal stress, was used for the effective stress in both parts of the failure criterion. That is, the maximum normal stress and its gradient were computed at the failure location to predict failure load.

## 6. Numerical Modeling and Predictions

**Figure 15:**
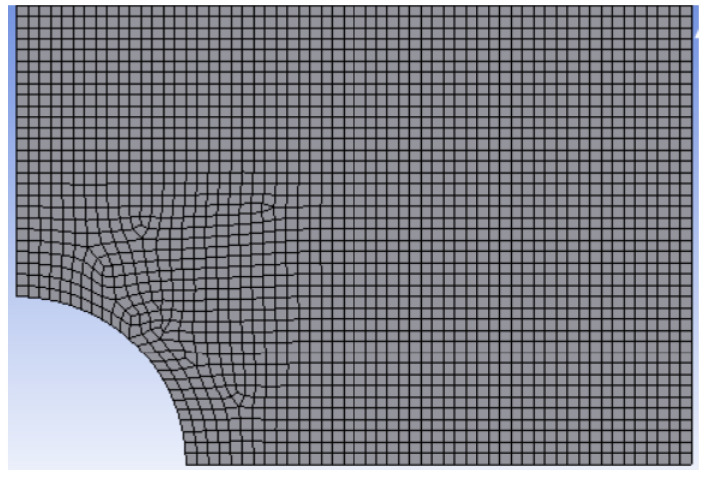
Finite element mesh around the hole.

To predict failure at the edge of each hole, FEA using the Ansys program was conducted to determine the maximum normal stresses and their stress gradients. Rectangular PLA specimens were modeled only for one-quarter of the geometry because of the presence of two symmetric planes. The symmetric boundary conditions were applied to the symmetric edges (i.e., left and bottom edges), and a uniform displacement was applied to the top edge to resemble the actual testing condition. Figure 15 shows the mesh around a quarter circular hole based on a study of mesh sensitivity for converged values of the stress concentration factor. Both uniform and nonuniform meshes were considered in the study. A typical mesh had approximately 50,000 four-node quadrilateral elements and 40,000 nodes. Because failure occurred along the minimum cross-section of every rectangular specimen, the stress gradient was computed along the failure direction. All the analyses were linear elastic using the elastic moduli as discussed previously.

**Figure 16:**
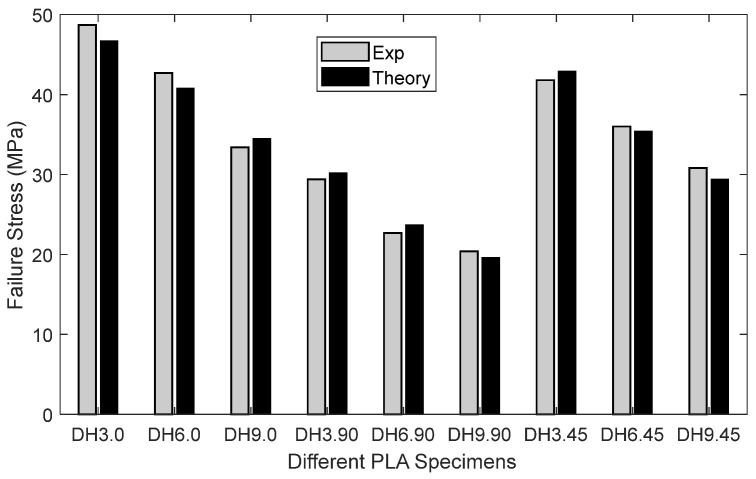
Experimental and theoretical failure stress for drilled PLA specimens.

As shown in Figure 13, the stress at which failure occurred was quite consistent for all specimens. Thus, the average value of the failure stresses was used for comparison to the analytical prediction. The failure stress in Figure 13 is the applied stress at the boundary where a uniform displacement was applied. It is not the stress at the edge of the hole. The comparison between experimental failure stress is compared to theoretical predictions, using the new two-part failure criterion, as shown below in Figure 16. Three different-sized holes were considered along with the three different printing angles. Only the drilled hole specimens were included because the rough edges would have been prohibitively difficult to model. Both failures compared very well with each other regardless of the hole size and printing angle. That is, the failure criterion [18,19,20] predicted applied failure stresses successfully for the PLA specimens.

Because PLA specimens had orthotropic material properties, different printing angles produced different material properties. That is, both stiffness and strength varied with the print angle. Thus, critical failure surface energy, $\kappa_{fail}$, also varied with the printing angle. This required determination of $\kappa_{fail}$ for every different printing angle. Those values were used in Figure 16 to predict the applied failure stress. To minimize having to repeat tests to obtain $\kappa_{fail}$ for each different angle, a semi-empirical equation was developed to predict the critical failure surface energy $\kappa_{fail}$ as a function of print angle for PLA specimens.

**Figure 17:**
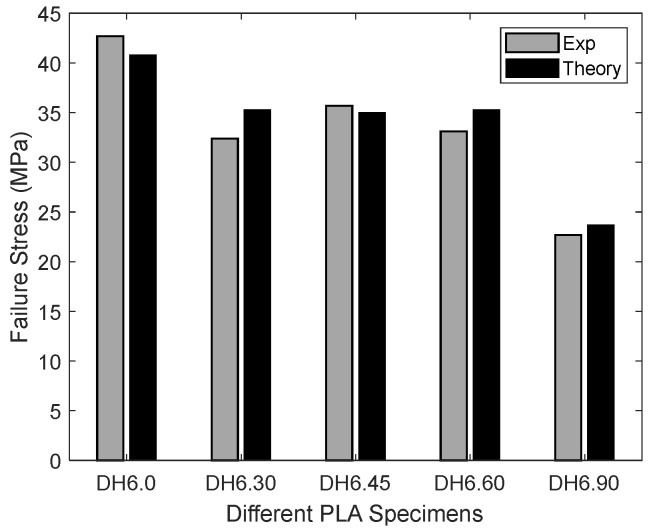
PLA failure stress for 6 mm drilled hole as a function of printing angle.

The semi-empirical equation for the failure surface energy is

(14) $${\left(\kappa_{fail}\right)}_{\theta }=\frac{{\left(\kappa_{fail}\right)}_{0}+{\left(\kappa_{fail}\right)}_{\pi /2}}{2}+\frac{{\left(\kappa_{fail}\right)}_{0}-{\left(\kappa_{fail}\right)}_{\pi /2}}{2}{\left(\cos2\theta \right)}^{7},$$

where *θ* is the printing angle. Once the critical failure surface energy was determined using Equation (14), the applied failure stresses were again predicted for different printing angles to assess the semi-empirical equation. The comparison for the samples with 6 mm holes can be seen below in Figure 17. Both experimental and theoretical applied failure stresses were in close agreement, which confirmed that the semi-empirical equation produced reliable critical failure surface energy.

**Figure 18:**
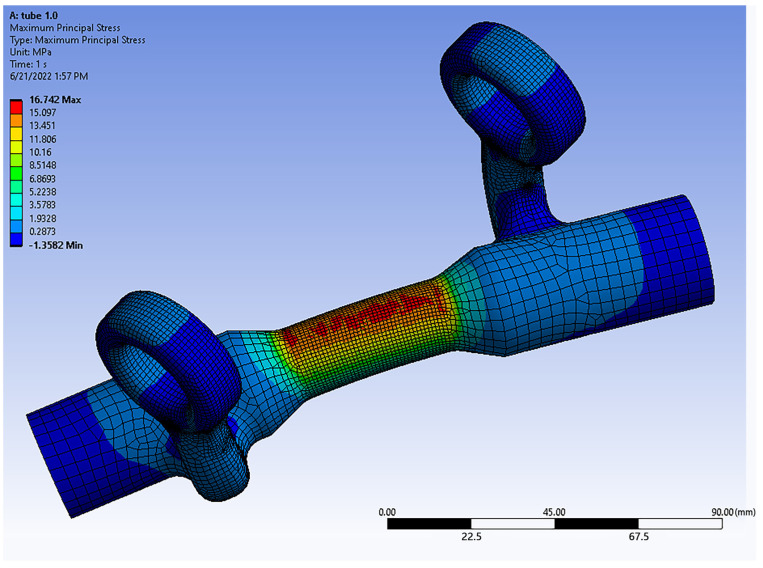
FEA of a combined loading specimen.

The next study was the combined loading of PC samples. These specimens were treated as isotropic due to the behavior exhibited previously and shown in Figure 14. A result from one of the combined loading scenario models is shown below in Figure 18. This FEA model included the whole specimens, as well as the upper and lower handles, as sketched in Figure 8. The bottom side of the lower handle was assumed to be fixed while the top side of the upper hand was loaded by external loading. Contact conditions were applied wherever the test specimen was in contact with the upper and lower handles. After a study of mesh sensitivity, about 200,000 eight-node solid elements were used based on the mesh sensitivity study.

As expected, this test section achieved the highest stress state. Four different kinds of specimens, previously seen in Figure 10, were constructed using the Ultimaker© S5 and Fortus© 450mc printers, respectively. As a result, in total eight different specimens were printed because both 3D printers produced different thicknesses. In addition, the infill percentage for the Fortus© 450mc could not be controlled because it was already set for the printer. Every specimen had two duplicates because each took a long time to print.

The original specimens had a 35.5 mm bending arm and a 32.5 mm torsional arm. The greater torsion specimens had a 52.5 mm torsional arm, while the bending arm was unchanged. The test section was 40 mm long. These dimensions were the same for both specimens regardless of the printer used.

**Figure 19:**
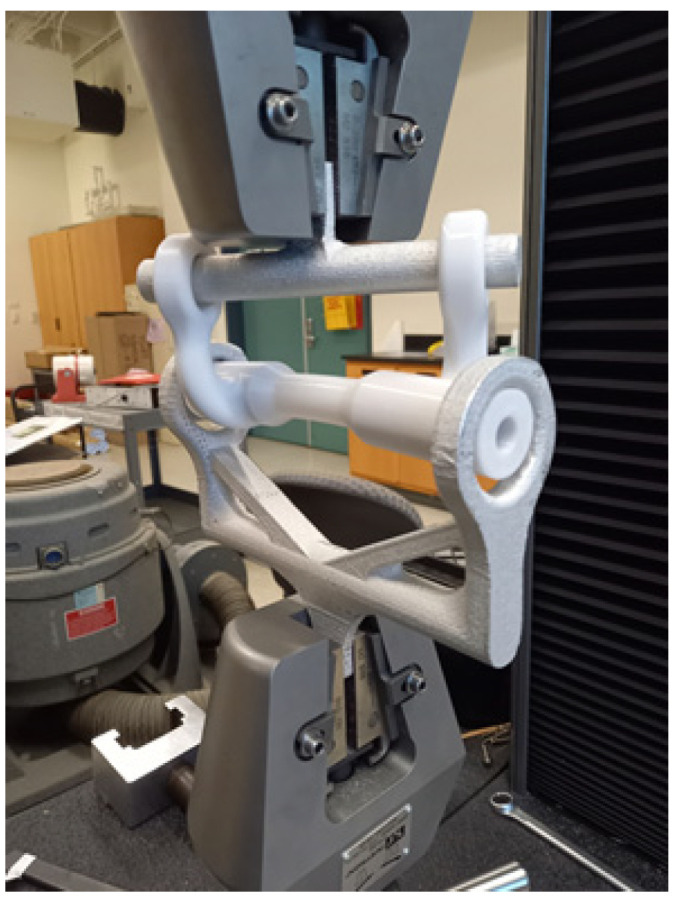
Combined loading specimen in the uniaxial test machine.

**Figure 20:**
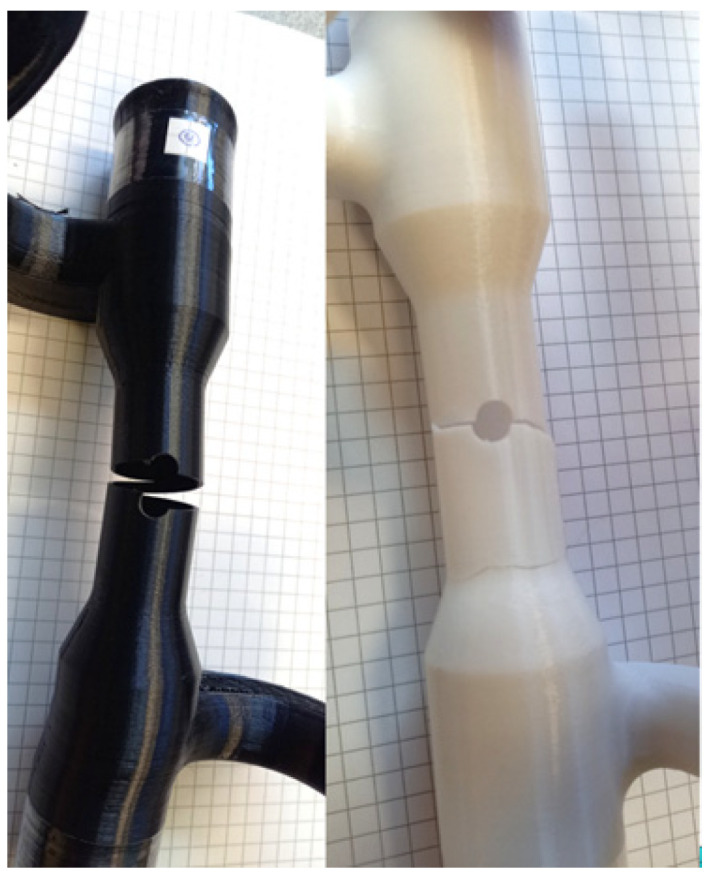
Post-test photograph of combined loading specimens (left specimen fabricated using Ultimaker© and right specimen fabricated using Fortus©).

Figure 19 shows a combined loading specimen being tested in the uniaxial test machine. All the combined loading samples were tested until fracture. Figure 20 shows the fracture across the hole. As expected, the failure location agreed well with the FEA results.

Before comparing the experimental failure stresses to analytical values, FEA results were compared to the simplified stress analysis for the combined loading tests using specimens without holes using Equations (1)–(6). Table 4 compares the maximum normal stresses determined from FEA and the analytical solutions using Equations (1)–(6). The same failure loads were applied to FEA as well as Equations (1)–(6) to obtain the maximum normal stresses.

The maximum normal stress varied from 6% to 20% between theory and FEA. One of the reasons for such differences was the torsional arms which were made of PC. Because the PC is more flexible than aluminum, the torsional arms deformed during the test. This would likely have affected the stress evolved in the test section and could have been one reason why the longer torsional arm resulted in a greater difference between the theory and FEA. In addition, torsional and bending arm lengths could not be precisely determined, as shown in Figure 8. Considering these differences, the analytical stresses were reasonable as compared to FEA.

Even though the analytical solutions are not very accurate as compared to the FEA solution, the analytical equations can be used in the designing process of the test setup and specimens without spending too much time on FEA.

Because there was no direct measurement of local stresses, failure stresses were computed using FEA as the experimental failure loads were applied to the FEA models. The failure stresses were the maximum normal stresses occurring in the specimens at the edge of the hole when the experimental failure loads were applied to the FEA model. To predict the failure stresses for the tubular specimens with a hole, Equations (12) and (13) were applied to every specimen. The stress gradient was computed using the FEA stresses at the two neighboring nodes along the failure direction starting from the edge of a hole. Then, an extrapolation technique was applied to find the stress gradient at the edge of the failure initiation site.

**Figure 21:**
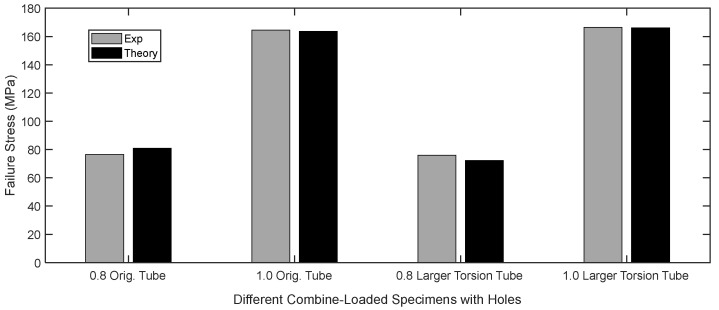
Comparison of the maximum stresses at the failure of combined loading specimens.

The results showed that Equation (13) is more critical than Equation (12). In other words, the failure stress from Equation (13) was greater than the failure stress from Equation (12). Because both parts of the criterion must be satisfied, the failure stress from Equation (13) was the predicted failure stress at the edge of the hole. The comparison between the experimental and analytical failure stress at the edge of the hole is shown in Figure 21. All the test specimens showed a good agreement between the theoretical and experimental failure stresses.

## 7. Summary and Discussion

Material properties of 3D printed PLA and PC specimens were experimentally measured using tensile testing. To avoid failure at the grip sections, test specimens used tabs at both ends. To simplify 3D printing, tabs were only printed on one side of each specimen so that no supporting material was needed. Print settings were chosen to induce orthotropic behavior in PLA specimens. Varying the angle of the printing direction using PLA resulted in behavior similar to that of a laminated fibrous composite. PC specimens, however, showed nearly isotropic behavior as the printing direction was changed.

Once obtaining material properties, a circular hole was introduced to PLA specimens which had different printing angles, while the overall specimen width, thickness, and length remained unchanged. These holes were made differently. In one scenario, the samples were printed with the hole; in the other, the hole was drilled out of the center of an otherwise pristine sample. The holes that were printed as part of the test specimens were rough and, as a result, induced a greater stress concentration. This manifested itself as a decreased failure load. Drilling the holes as the specimens were fully supported at their backsides, however, resulted in much smoother edges and a higher failure stress than that exhibited by the former samples. Using the samples with the printed holes was demonstrated to influence the failure load significantly; because of the lack of consistency and computational complexity in modeling the rough hole edges, they were not used in the study. Instead, the printed holes were considered for modeling and comparison.

In addition to flat PLA samples, tubular PC specimens were tested but under different loading conditions. These samples were subjected to combined loading using a specially designed test specimen geometry which could be tested using ordinary uniaxial test equipment. The test sample consisted of three unique parts, designed and fabricated using a 3D printing technique to produce complex shapes. The combined loading used to test these samples consisted of both bending and torsion. The magnitude of applied bending and torsional moments was controlled by varying the moment arm of each loading in the test setup. Stresses resulting from combined loading without stress concentrations were computed using the mechanics of materials approach, and the analytical stresses were in reasonable agreement with those calculated from FEA. The difference was due to the flexibility of the torsional arm of the tubular specimens, as well as an ambiguity in precisely defining the length of the moment arms. Both could be improved by modifying the design, although neither was attempted here since this was not the main focus of this study. Instead, the test setup was modeled using FEA to predict the resultant stresses occurring in the test specimens.

The failure loads of both PLA and PC specimens with a hole were predicted using a new failure criterion which used the stress gradient at the failure site. Maximum normal stresses were used for the failure criterion. The stress gradient was subsequently calculated using the slope of maximum normal stresses at the edge of the hole along the failure direction.

The PLA tensile specimens had three different hole diameters, while their width and length remained the same. In addition, the print angle was varied for the PLA specimens such as +q°/−q°, and q changed from 0° to 90° in fixed increments. There were nine different PLA specimens with a hole. At the loading boundary, the average applied stresses were calculated from the failure criterion and experimental data, and they were found to agree very well with each other.

The stress gradient-based criterion requires a material constant called the critical surface failure energy. When the printing angle was changed for the PLA samples, the critical failure surface energy was found to change as a result, similar to how laminated fibrous composites behave. To minimize redundant testing to determine the critical failure surface energy, a semi-empirical equation was developed to predict it for different printing angles. The failure stresses obtained using the critical failure surface energy predicted from the semi-empirical equation also agreed well with the experimental results. This confirmed that the semi-empirical equation was useful to estimate the critical failure surface energy.

The PC tubular specimens with a hole were also compared for their failure stresses between the analytical and experimental results. The failure stresses were the maximum normal stresses at the failure location of the holes as the specimens were subjected to combined loading which consisted of bending and torsion. For experimental results, the applied loads at failure were used in FEA to determine the maximum normal stresses at the failure locations because there was no direct measurement of local stresses during tests. There were four different tubular PC specimens with holes, with two different torsional arms printed using two different printers. The predicted failure stresses, i.e., maximum normal stresses, at the hole edges were in good agreement with the experimental data.

## 8. Concluding Remarks

A new failure criterion was used to predict failure loads and stresses for different types of 3D printed specimens with holes subjected to uniaxial and combined loading. To apply combined loading consisting of bending and torsion using uniaxial testing equipment, a novel test setup and testing specimens were designed and fabricated by taking advantage of flexibility in AM.

When failure stresses were calculated using these new criteria, they were found to be in good agreement with experimentally determined values for all the test specimens. As a result, this new criterion can be used to design a wide variety of structural parts with holes without the need for repetitive testing. This criterion was also useful for assessing the failure stress of 3D printed parts, which were printed in such a manner that their strength was found to vary as a function of printing angle.

Lastly, a semi-empirical equation was proposed to estimate the critical failure surface energy for different printing angles in parts, which is necessary to apply the new failure criterion for the prediction of failure loads and stresses. The equation was validated against additional experimental data. This equation can also help to eliminate additional tests to determine new material properties with different printing angles in designed parts.

## Figures and Tables

**Table 1:** PLA sample print settings.

Print Temperature	185 °C
Bed temperature	55 °C
Print speed	45 mm/s
Layer height	0.2 mm
Line width	0.35 mm

**Table 2:** Print settings for Ultimaker© printing PC.

Print Temperature	280 °C
Bed temperature	120 °C
Print speed	45 mm/s
Layer height	0.2 mm
Line width	0.4 mm
Brim line count	10

**Table 3:** Properties of PLA specimens.

E_1_	2.33 GPa
E_2_	2.14 GPa
G_12_	1.04 GPa
n_12_	0.375
(s_1_)_fail_	57.7 MPa
(s_2_)_fail_	23.3 MPa

**Table 4:** Comparison of maximum normal stresses of tubular specimens subjected to combined loading.

	Thickness	σ_max_ (Analytic)	σ_max_ (FEA)	Error
Original With no hole	0.8 mm	44.18 MPa	46.85 MPa	6.04%
	1.0 mm	66.23 MPa	71.62 MPa	8.14%
Longer torsional arm With no hole	0.8 mm	49.20 MPa	54.70 MPa	11.19%
	1.0 mm	69.19 MPa	82.56 MPa	19.33%

## Data Availability

All the data are provided in this paper in the form of figures and tables.
